# Risk Factor Scenario in an Industrial Set-up: Need for an Effective Screening Tool to Assess the High-Risk Group

**DOI:** 10.4103/0970-0218.66884

**Published:** 2010-04

**Authors:** Uma Iyer, Garima Mathur, Nandini Panchanmiya, Swati Dhruv

**Affiliations:** Department of Foods and Nutrition, Faculty of Family and Community Sciences, The Maharaja Sayajirao University of Baroda, Vadodara, Gujarat, India

**Keywords:** Industry, risk factors, prevalence of non-communicable diseases, lipid profile

## Abstract

**Background:**

Industrial and technological revolution has resulted in nutrition transition. This calls for analyzing the risk factor scenario in the industrial population.

**Objective:**

The objective was to map the prevalence and assess the risk factors of industrial employees.

**Materials and Methods:**

The employees of a large petrochemical industry were enrolled (*N*=269) for the study. Risk factors were elicited through a structured questionnaire. Parameters monitored were fasting blood sugar and lipid profile. Relative risk was calculated to find out significant predictor variables.

**Results:**

The employees had high prevalence of overweight (27%), obesity (22%), central obesity (48.7%), prehypertension (43.2%), hypertension (36.6%), and dyslipidemia (41.4%). They had erroneous dietary habits such as low intake of fruits and vegetables and high fat intake. Most of the employees had low physical activity levels. The prevalence of smoking (13.5%), tobacco (28.2%), and alcohol use (22.2%) were also high with 15.1% having multiple habits. One-fifth of the employees had metabolic syndrome (MS). Seven predictor variables, namely, family history, BMI, WHR, blood pressure, physical inactivity, TG, and TG/H were identified and used to develop the risk score card to identify people at high risk of CVD and DM.

**Conclusion:**

Multiple risk factor scenario among the industrial population studied calls for effective intervention strategies and policy changes to combat the burden of non-communicable diseases. The risk score card can be used to screen the high-risk group in the industrial population.

## Introduction

Non-communicable diseases (NCDs) are becoming increasingly significant causes of disability and premature death in both developing and newly developed countries. The rapid transition in the food habits, lifestyle, and the ethnic differences are the major factors which have contributed to this. The disease burden has also shifted from the older age group to the more productive middle age group.(1) Both preventive and corrective measures are required to reduce the disease burden and ensure a healthy and productive workforce. According to the World Health Report, 2003,(1) NCDs caused 6.08 million deaths of which cardiovascular diseases (CVDs) alone caused 3.31 million deaths in the South East Asian region in 2002. Age, sex, ethnicity, family history, stress, dyslipidemia, high blood pressure, obesity, physical inactivity, and insufficient consumption of fruits and vegetables together with alcohol and smoking are the risk factors for NCDs.(2–6) Demographic and health transitions, gene-environment interactions, and early life influences of fetal malnutrition have been implicated as causes of the increasing burden in India.(7) Dietary deficits and excesses and the lifestyle changes that accompany the economic development make a significant contribution to this epidemic. NCDs alone resulted in 125.5 million DALYs (disability adjusted life years) lost as compared to 39.7 million due to injuries in India.(8) The unbalanced diet and increasing physical inactivity clubbed with a stressful routine lead to increase in the incidence of NCDs and a decrease in the work efficiency and productivity. With this background, the present study was planned to address the following: (a) The magnitude of the problem in a petrochemical industry. (b) The identification of predictors of CVDs and diabetes.

## Materials and Methods

A large petrochemical industry located on the outskirts of Vadodara was purposely selected for the study. It had 10 working plants from which 2 were selected purposely. The employees were enrolled on the basis of voluntary participation (*N*=269). Information was collected regarding the general background, medical history, lifestyle factors, anthropometric measurements, and dietary consumption pattern (type of diet, frequency of consumption of fruits and vegetables, dietary pattern on shift duty and holidays, packed lunch details for 2 days, consumption of foods in canteen). The anthropometric measurements used were body mass index (BMI), waist circumference (WC), waist hip ratio (WHR), and hip circumference (HC); per cent body fat was calculated using an online calculator for the employees.(9) Medical history was collected on the type of NCDs or any other medical condition the employees were suffering from. The duration of the disease and the age of diagnosis along with the family history of the diseases were also taken. Information was taken on personal habits like smoking, tobacco chewing and frequency of alcohol consumption; their exercise routine (if any); and their dietary habits. Biochemical estimations were done at the medical center of the industry. The lipid parameters, namely, total cholesterol (TC), triglycerides (TG), HDL cholesterol, and LDL cholesterol were estimated using the auto-analyzer (Bayer’s diagnostic, *n*=229). The blood pressure was noted by the medical officer using the standard procedure with the help of a sphygmomanometer. Metabolic syndrome is a constellation of metabolic and non-metabolic disorders related to defects in insulin sensitivity that leads to the development of type 2 diabetes and CVDs. In the present study, we have used the IDF criteria to map the prevalence of metabolic syndrome.

The data analysis were done with the MS excel statistical analysis package. Mean and standard deviation were calculated and student’s t-test was done to calculate the difference between groups. Relative risk was calculated using the “EPI-INFO” software package, to identify the risk factors. The tests were considered as significant at P0 ≤ 0.05.

## Results

Majority of the industrial population were Hindus (93.7%), lived in nuclear families (68.9%), belonged to the age group of 41-50 years (53.3%) with a mean age of 45.3 years, worked in rotating shifts (57.3%), and had been working in the industry for ≥20 years (62.6%).

The prevalence of obesity assessed by BMI using the Asia-Pacific classification was observed to be 21.7% (BMI ≥25) and for overweight (BMI 23–24.9), it was 26.8%. According to the WHO classification for the Asian population, 42.5% of the employees were overweight and 5.9% obese. About 4.7% of the employees were found to be underweight. According to the International Diabetes Federation criteria, central obesity was present in 48.7% of the employees while the prevalence was only 7.3% with the ATP III classification. Only 12.5% had WC <80 cm and about 38.8% had WC between 80 and 89 cm. Using the WHO criteria of WHR, it was observed that 68.7% of the employees had central obesity. The prevalence of obesity according to per cent body fat revealed that 68.7% had ≥25% body fat indicating obesity. Around 25.1% had body fat in the range of 18–24.9% which is acceptable but above the fitness levels.

The prevalence of hypertension, diabetes, and CHD based on medical history was found to be 14.2%, 6.4%, and 0.4%, respectively, among the employees. Hypertension was the predominant clinical condition. Out of the total employees enrolled, blood pressure measurements could be obtained for 229 employees. Using the JNC VII classification, it was observed that 48.2% were in the prehypertensive stage and 36.7% had either systolic or diastolic blood pressure above normal. Only 20% of the employees had normal blood pressure values. Around 21% had history of other clinical conditions like joint pain, gout, asthma, etc.

Regarding family history of NCDs, about 14.7% had parental history and 3.8% had sibling history of hypertension. Parents of 8.6% of the employees had diabetes and 6% of the employees had parental history of both diabetes and hypertension. Around 46% and 18% of the employees had a positive family history with regard to parents and siblings, respectively, for CHD and/or hypertension and/or diabetes.

When the individual data with respect to lipid profile were looked into, a high prevalence of dyslipidemia was seen [Table 1]. The prevalence of hypercholesterolemia, hypertriglyceridemia, and elevated levels of atherogenic lipoprotein (LDL) was seen in 41.4%, 32.7%, and 85.6%, respectively, of the employees. The TG/H ratio, which is an indicator of small dense lipoprotein and is regarded as an index to mark dyslipidemia, was found to be high in 42.4% of the employees.

**Table 1:** Prevalence of dyslipidemia in the employees

Parameters	Percentage
TC	41.5
TG	32.7
HDL-C	20
LDL-C	85.6
VLDL-C	32.8
NON-HDL-C	30.6
TC/H	18.8
L/H	55.9
TG/H	42.4

The prevalence of metabolic syndrome which signifies the clustering of risk factors was found to be higher (18.7%) when IDF criteria were used as compared to the ATP III (13.9%) criteria.

With regard to lifestyle factors, about 13.5% of the employees were smokers. Alcohol was consumed by 22.2% and tobacco chewing was prevalent in 28.2% of the employees. It was observed that more than one-tenth of the employees (15.1%) had multiple habits of smoking and/or tobacco chewing and/or consuming alcohol. Around 16.2%, 11.3%, and 7.5% had past history of smoking, tobacco chewing, and alcohol consumption, respectively. The self-reported physical activity was below the recommended levels of ≥3 h/week in 48.5% of the subjects. The intake of fruits was reported to be low. Around 75% of the employees did not consume fruits on a daily basis. The consumption of citrus fruits was poor with 61.6% of the employees not consuming them or eating citrus fruits only occasionally. Further 63.7% of the employees rarely ate more than one kind of fruit at a given time. Daily intake of green leafy vegetables (GLVs) was also low (5.7%) in the employees. Around 40.7% consumed GLVs one to two times a week. About 14.3% reported that they consumed GLVs more than three times per week during the winter season. Starchy roots, mainly potato, were consumed by majority of the people (49.3%) daily and 22.3% had it more than three times a week.

With regard to predictive variables, the relative risk for 27 variables was calculated for the 207 employees on whom all the data were available and were looked into in relation to clinical condition (CHD, hypertension, diabetes mellitus). The predictor variables identified on the basis of significance at 95% confidence interval limits were family history, presence of overweight/obesity, WHR, PA, hypertension, TG, VLDL, and TG/H ratio [Table 2]. An attempt was made to identify the high-risk group with a simple risk score card in the industrial population using a composite risk scoring system [Table 3]. Five points were given to each risk factor identified (for ease of calculation at field level) and a simple risk score card was developed with seven variables.

**Table 2:** Relative risk values for various risk factors

Variable	Relative risk	Range	*P* value
Only parent history	2.22**	1.45<RR<3.38	0.0002
Only sibling history	2.65*	1.57<RR<4.46	0.020
Either sibling or parent history	2.72***	1.74<RR<4.25	0.0000045
Anthropometric measures			
BMI ≥ 23	0.72**	0.54<RR<0.97	0.01
WC ≥ 90 cm	1.09	0.71<RR<1.66	0.705
WHR ≥ 0.9	3.07 ***	1.55<RR<6.09	0.00024
WC/weight >1.36	0.78	0.51<RR<1.18	0.245
Biophysical parameters			
Blood pressure ≥140/ ≥90	1.91 **	1.25<RR<2.9	0.0027
Lifestyle factors			
Physical inactivity	1.64 *	1.05<RR<2.11	0.026
Smoking	1.24	0.71<RR<2.15	0.4658
Alcohol use	0.73	0.41<RR<1.29	0.2658
Tobacco use	0.86	0.53<RR<1.4	0.540
Dietary factors			
Low citrus fruits’ intake	0.78	0.41<RR<1.49	0.432
Low GLVs’ intake	1.73	0.76<RR<3.97	0.362
Biochemical indicators			
High TC ≥ 200 mg/dl	1.38	0.9<RR<2.11	0.141
High TG ≥150 mg/dl	1.86 **	1.23<RR<2.01	0.0043
High LDL ≥ 100 mg/dl	0.94	0.54<RR<1.64	0.83
Low HDL <40 mg/dl	1.18	0.72<RR<1.95	0.523
High non-HDL ≥ 150 mg/dl	1.26	0.81<RR<1.97	0.31
High VLDL >30 mg/dl	1.96 **	1.3<RR<2.96	0.0019
High TC/H ≥ 5	0.98	0.56<RR<1.73	0.95
High L/H ≥ 2.5	1.02	0.66<RR<1.58	0.91
High TG/H ≥ 3	1.59 *	1.04<RR<2.43	0.03

**Table 3:** Mean scores of the employees with and without clinical conditions

Without clinical condition N=111	Clinical condition *N*=53	Total*N*=207	*t* value
13.9 ± 7.88	20 ± 7	16.47 ± 8.06	9.51***

Taking the standard deviation and number of risk factors into consideration, the following cut-off values were arrived at: ≤10-low risk, 10-20-moderate risk, and ≥20-high risk.

Overall 46% of the employees were in high risk and 21% in low risk. An important fact which emerged was that in non-clinical cases, there were only 46% of the employees in the low-risk category. If one looks at the numbers of risk factors present which are given in Table 4, it was found that 46% of the employees had more than or equal to four risk factors.

**Table 4:** Overview of the risk factors present in the industrial employees

	Total
N	207
No RF	7 (3.38)
1 RF	24 (11.6)
2 RF	37 (17.9)
3 RF	44 (21.3)
≥ 4 RF	95 (45.9)

Out of total 269 subjects, the data were available for 207 subjects and hence final *N*=207

## Discussion

In the present study, the medical history revealed the prevalence of hypertension to be 14.2%. When the clinical profiles were looked into, it was seen that 36.7% had hypertension (JNC VII classification) and 48.2% were in the prehypertension stage indicating a large population at risk. Thus, our findings highlight the need to monitor blood pressure regularly and take early preventive steps. Around 46% of the employees had a positive parental history of CVDs and/or DM and/or hypertension. The relative risk was 2.72 (*P*<0.001) in employees having parental and/or sibling history of NCDs.

Obesity is associated with increased morbidity and mortality by influencing lipid levels, blood pressure, glucose tolerance, and inflammatory markers. The high prevalence of obesity observed in the study population emphasizes the need for regular screening and taking preventive steps as early as possible to delay the onset or totally prevent the development of NCDs. Independent of age, WC contributes to the prediction of non-abdominal, abdominal subcutaneous, and visceral fat.(10) This underscores the importance of incorporating WC into routine clinical practice to identify those at increased health risk. In the present study, the routine medical examination did not include WC measurements.

Physical inactivity was found to be high (23.3%) in the present study, in contrast to 3.4% as reported in the sentinel survey. This reveals the fact that physical activity levels are low in the industrial population and there is a need to encourage employees to engage in more vigorous activities. About 22.2% of the employees of the petrochemical industry belonged to the category of current alcohol users. Since in the present study, it was mandatory not to smoke around the worksite due to safety requirements, smoking was partially or wholly substituted by tobacco chewing. Therefore, the prevalence of tobacco chewing was found to be 28.2% which was much higher as compared to 9% found in the sentinel survey.

Low fruit and vegetable intake is among the top 10 risk factors contributing to the attributable mortality.(1) Daily intake of green leafy vegetables was reported to be poor among the employees, and majority of the employees (75%) did not have fruits daily. The industrial canteen provided fried snacks at a subsidized cost encouraging the employees to have it on a daily basis. Further, the practice of taking the food home increases the trans fatty acid intake of the other family members.

High levels of atherogenic indicators like TG/H and L/H were also found in 42.4% and 55.9%, respectively, of the industrial population. This highlights the fact that dyslipidemia may predispose the individuals to cardiometabolic risk. This is further substantiated by the high prevalence of metabolic syndrome.

The observations of the industrial population studied are similar to the sentinel study done in 10 industries of India.(7) A number of risk factors were looked into and the predictors of CVDs and DM were identified. In all, the seven factors which came out significant were family history, BMI, WHR, blood pressure, physical inactivity, TG, and TG/H. These factors were used to develop a simple risk score card to identify the high-risk group. The tool developed needs to be tested for its sensitivity and specificity.

Since the worksite is a place regularly visited by an individual, it is the most appropriate place to help reduce the future prevalence and incidence of chronic diseases. It is in the employer’s best interest to take necessary measures in order to detect the presence of risk factors and plan effective interventions to reduce direct as well as indirect costs to the company and ensure better productivity.

## Conclusions

The simple risk score card developed should be internalized with the routine yearly medical examinations of the employees to screen the high-risk group and to monitor the progress related to risk factors. The high-risk group can be focused for targeted intervention. Further, a nutrition health index card can be given to the employees to make them aware of their health risk status and can also be used as a reference for follow-up during the subsequent annual check-up.
